# Complete Genome Sequence of the Clinical Beijing-Like Strain *Mycobacterium tuberculosis* 323 Using the PacBio Real-Time Sequencing Platform

**DOI:** 10.1128/genomeA.00371-15

**Published:** 2015-04-30

**Authors:** Juan Germán Rodríguez, Camilo Pino, Andreas Tauch, Martha Isabel Murcia

**Affiliations:** a Departamento de Microbiología, Facultad de Medicina, Grupo MICOBAC-UN, Universidad Nacional de Colombia, Sede Bogotá, Colombia; b Facultad de Ingeniería, Grupo BioLISI, Universidad Nacional de Colombia, Sede Bogotá, Colombia; c Institut für Genomforschung und Systembiologie, Centrum für Biotechnology (CeBiTec), Universität Bielefeld, Bielefeld, Germany

## Abstract

We report here the whole-genome sequence of the multidrug-resistant Beijing-like strain *Mycobacterium tuberculosis* 323, isolated from a 15-year-old female patient who died shortly after the initiation of second-line drug treatment. This strain is representative of the Beijing-like isolates from Colombia, where this lineage is becoming a public health concern.

## GENOME ANNOUNCEMENT

In recent years, strains of the *Mycobacterium tuberculosis* Beijing lineage have become successful pathogens worldwide due to their capacity to spread in a population. In some cases, these strains have been associated with multidrug-resistant tuberculosis outbreaks (1). In Colombia, the Beijing lineage has been reported since 1998 (2), and recent evidence suggests that strains of this lineage have an increased incidence and are associated with multidrug-resistant tuberculosis outbreaks (3, 4). We therefore sequenced the complete genome of an *M. tuberculosis* Beijing-like strain with the internal nomenclature isolate 323 to gain insights into its genetic characteristics and develop new diagnostic tools to improve the epidemiological control of this dangerous *M. tuberculosis* lineage.

Spoligotyping was used to confirm the assignment of isolate 323 to the Beijing-like lineage of *M. tuberculosis* (5). It was classified as spoligotype international type 190 (SIT190), as previously reported (3). Genomic DNA was extracted from isolate 323, according to the protocol provided by the Institute for Genomic Sciences, University of Maryland (standard operating procedures [SOP] manual 26, version 2.01). The DNA was sequenced using the PacBio RS II sequencer. The hierarchical genome assembly process from the Pacific Biosciences single-molecule real-time (SMRT) Analysis tool kit was used (6) to obtain a *de novo* assembly. The original reads were aligned to the trimmed assembly (7) using BLASR (8). The results showed an assembly with a single contig and a sequence length of 4,411,216 bp, with an average coverage depth of 298.65×. The *N*_50_ contig length was 8,627 bp, and the mean read length was 5,971 bp. The assembled sequence was annotated using the GenDB annotation system for prokaryotic genomes (9). The genome sequence has a mean G+C content of 65.6%, 4,237 protein-coding regions, 45 tRNA genes, and 3 rRNA genes. Taking the genome of *M. tuberculosis* H37Rv as a reference sequence (10), the assembly has an average identity of 99.88%, with 3,507 total variants, comprising 2,143 single nucleotide polymorphisms (SNPs), 207 multiple nucleotide polymorphisms, 588 insertions, and 569 deletions. Among the SNPs, 1,787 were detected in coding regions, of which 743 are synonymous, 1,026 are missense, and 18 are nonsense substitutions.

SNPs known to confer resistance to rifampin (S450L in *rpoB*), isoniazid (S315T and R463L in *katG* and D229G in *accD6*), ethambutol (M306V in *embB*), pyrazinamide (T142A in *pncA*), and streptomycin (K43R in *rpsL* and E92D in *gidB*) were observed and confirm the multidrug-resistant phenotype of isolate 323. Moreover, a novel mutation was observed in the *embC* gene (deletion of C2972) coding for ethambutol resistance. Regarding second-line antituberculous drugs, a known mutation in the *gyrA* gene (D94G) and the novel mutations E21Q and D668G were detected. SNPs known to confer resistant to amikacin/kanamycin/capreomycin (A1401G in *rrs)* and a novel mutation in the *eis* gene (deletion of CA804) were observed, suggesting that this strain is extensively drug resistant. This is the first report of whole-genome sequencing of a multidrug-resistant *M. tuberculosis* Beijing-like isolate circulating in Colombia. With this genome project, we demonstrate that PacBio sequencing technology is a reliable method for generating finished microbial genomes of *M. tuberculosis* isolates.

### Nucleotide sequence accession number.

The genome sequence of the *M. tuberculosis* Beijing-like isolate 323 has been deposited in GenBank under the accession no. CP010873.
